# Supplementary material for: Onward Spread from Liver Metastases Is a Major Cause of Multi-Organ Metastasis in a Mouse Model of Metastatic Colon Cancer
Source: Cancers (Basel). 2024 Mar 6;16(5):1073. doi: 10.3390/cancers16051073 (PMC10930793; doi:10.3390/cancers16051073)
Supplement: Supplementary file 1 [file cancers-16-01073-s001.zip › cancers-2839333-supplementary.pdf]

Supplemental Video S1: Liver implantation

Supplemental Video S1: Caecum implantation

Supplemental Table S1: Summary of APC stop-gained and SMAD stop-gained and frameshift mutations in Mouse Tumor Derived Organoids (MTDO)

Gene Name	*Annotation*	*Putative_impact*	*HGVS.c*	*HGVS.p*
Apc	stop_gained	HIGH	c.3578C>A	p.Ser1193*
Smad4	frameshift_variant	HIGH	c.306_307insCCGA	p.Asp103fs
Smad4	synonymous_variant	LOW	c.306T>A	p.Pro102Pro
Smad4	stop_gained	HIGH	c.303G>A	p.Trp101*
Smad4	missense_variant	MODERATE	c.301T>C	p.Trp101Arg
Smad4	frameshift_variant	HIGH	c.288_298delCCGTCTGTGGA	p.Arg97fs
Smad4	conservative_inframe_insertion	MODERATE	c.286_287insATA	p.Ala96delinsAspThr
Smad4	frameshift_variant&stop_gained	HIGH	c.283_284insTTTA	p.Tyr95fs
Smad4	synonymous_variant	LOW	c.282C>T	p.Ile94Ile

Supplemental Table S2: Metastastic efficiency is higher from liver tumors than caecum tumors. A population based generalized estimating equations (GEE) model was used to compare metastatic efficiency of MTDO4 between liver (n=44) and caecum implantation (n=16) models.

Metastastic efficiency is higher from liver tumors than caecum tumors.
A population based generalized estimating equations (GEE) model was used to compare metastatic efficiency of MTDO4 between liver (n=44) and caecum implantation (n=16) models.

		Mean Difference (I-J)	Std. Error	df	Sig.	95% Wald Confidence Interval for Difference	
						lower	upper
Caecum_primary tumor	Liver_ primary tumor	-0.69	0.067	1	0.000	-0.82	-0.56
Caecum_liver Metastasis*	Liver_ liver metastasis*	-0.25	0.073	1	0.001	-0.39	-0.1
Caecum_lung metastasis	Liver_lung metastasis	-0.13	0.055	1	0.017	-0.24	-0.02
Caecum_peritoneal metastasis	Liver_peritoneal metastasis	-0.45	0.141	1	0.001	-0.72	-0.17

*spread to distant liver lobes other than primary liver tumor

Supplemental Table S3: Differentially expressed immune cell signatures and metabolic pathways in liver metastases *versus* primary tumors.

**Hallmark pathway**	**MIN-LOG10 p-value**	**higher in**	**p-value**
xenobiotic metabolism	2,28	liver metastases	0,0052
bile acid metabolism	1,85	liver metastases	0,014
**Immune cell signatures**			
B cells	1,68	primary tumor	0,021
T helper 17	1,64	primary tumor	0,023
mast cells	1,33	primary tumor	0,047
T helper cells	1,34	primary tumor	0,046
